# Mycobacterial phosphatase PstP regulates global serine threonine phosphorylation and cell division

**DOI:** 10.1038/s41598-019-44841-9

**Published:** 2019-06-06

**Authors:** Iswahyudi, Galina V. Mukamolova, Anna A. Straatman-Iwanowska, Natalie Allcock, Paul Ajuh, Obolbek Turapov, Helen M. O’Hare

**Affiliations:** 1 Leicester TB Research Group, Department of Respiratory Sciences, University of Leicester, LE1 7RH Leicester, UK; 2 Pharmaceutical Biology Department, Faculty of Pharmacy, Tanjungpura University, Pontianak, Indonesia; 3 Leicester Core Biotechnology Services Electron Microscopy Facility, University of Leicester, Leicester, UK; 4 Gemini Biosciences Ltd, Liverpool Science Park, Liverpool, United Kingdom; 5 Leicester Institute of Structural and Chemical Biology, Department of Molecular and Cell Biology, University of Leicester, Leicester, UK

**Keywords:** Kinases, Bacterial physiology, Proteomics

## Abstract

Protein phosphatase PstP is conserved throughout the Actinobacteria in a genetic locus related to cell wall synthesis and cell division. In many Actinobacteria it is the sole annotated serine threonine protein phosphatase to counter the activity of multiple serine threonine protein kinases. We used transcriptional knockdown, electron microscopy and comparative phosphoproteomics to investigate the putative dual functions of PstP as a specific regulator of cell division and as a global regulator of protein phosphorylation. Comparative phosphoproteomics in the early stages of PstP depletion showed hyperphosphorylation of protein kinases and their substrates, confirming PstP as a negative regulator of kinase activity and global serine and threonine phosphorylation. Analysis of the 838 phosphorylation sites that changed significantly, suggested that PstP may regulate diverse phosphoproteins, preferentially at phosphothreonine near acidic residues, near the protein termini, and within membrane associated proteins. Increased phosphorylation of the activation loop of protein kinase B (PknB) and of the essential PknB substrate CwlM offer possible explanations for the requirement for *pstP* for growth and for cell wall defects when PstP was depleted.

## Introduction

Serine/threonine phosphorylation in bacteria is a ubiquitous reversible signal that controls cell cycle events, virulence, and responses to environmental cues^1^. Phosphoserine and phosphothreonine are chemically stable, and the transient, reversible nature of the signal relies on the activity of protein phosphatases as well as protein turnover. The conserved Actinobacterial regulator GarA is an example of a stable phosphoprotein that is replaced by protein turnover since its buried phosphothreonine resists phosphatase activity^2^, while the autophosphorylation sites in the activation loops of protein kinases are examples of short-lived phosphorylation signals, since these exposed sites are dephosphorylated rapidly by protein phosphatase^3,4^.

Serine threonine (S/T) phosphatases are greatly outnumbered by S/T kinases in bacterial and eukaryotic genomes. Human protein phosphatases tend to have lower substrate specificity than protein kinases, and more substrates. Substrate selection is driven by subcellular localisation and interaction with regulatory partners^5,6^. Studies of purified bacterial S/T phosphatases suggest that these are also multi-specific^1,7^, for example recombinant *Mycobacterium tuberculosis* PstP dephosphorylates itself, protein kinases PknB and PknA and phosphoproteins PbpA and GarA^3,4,8^. These *in vitro* studies necessarily lack any regulatory proteins and use non-physiological protein concentrations. Phosphatase knockout in *Enterococcus faecium* has provided evidence of substrate selectivity in cells^9^.

**Figure 1:**
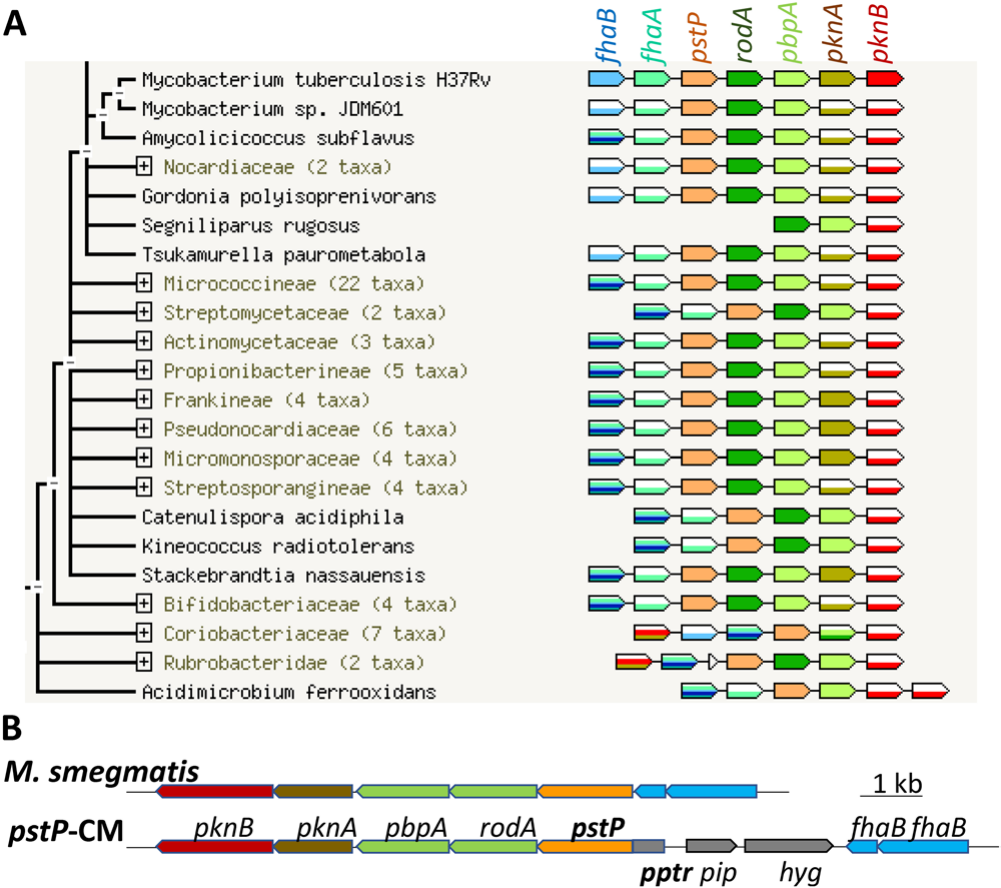
The *pstP* operon is conserved in the Actinobacteria, and was disrupted in this study to make a conditional mutant strain of *M. smegmatis*, *pstP*-CM. (**A**) PstP is encoded in a cluster of seven genes that are related to cell wall synthesis and protein phosphorylation. The genetic linkage of *pstP*, *rodA*, penicillin binding protein A (*PbpA*), and PASTA-kinase is conserved throughout the Actinobacteria. (**B**) To generate *pstP*-CM, homologous recombination was used to replace the endogenous *pstP* promoter with the pristinamycin-inducible promoter *pptr* and add the transcriptional repressor *pip*. In the absence of the inducer pristinamycin, Pip binds to *pptr* to repress transcription.

The ratio of S/T kinase to S/T phosphatase genes is particularly high in Actinobacterial genomes. Non-pathogenic *M. smegmatis* has 18 S/T kinases, one transmembrane and one soluble S/T phosphatase. The function of the soluble S/T phosphatase MspP (MSMEG_1928) is unknown, although its activity has been demonstrated *in vitro*^10^. Only the transmembrane S/T phosphatase PstP is conserved between *M. smegmatis* and *M. tuberculosis* (91% identity in the phosphatase domain), and this genetic locus is conserved throughout the Actinobacteria (Fig. 1) and is associated with cell division^11^. *pstP* is cotranscribed with genes involved in peptidoglycan synthesis *rodA* and *pbpA*^12,13^ and there is weak positive correlation of transcription with protein kinases *pknA* and *pknB*^14,15^. The kinase genes have separate transcriptional start sites^16^ and regulate cell growth and cell division^17^. *fhaA* and *fhaB*, also in this locus, encode phosphothreonine recognition proteins that regulate cell growth and cell division^18,19^.

The gene cluster *pknB-fhaA* is conserved in a subset of the Firmicutes as well as the Actinobacteria, while the link between *pstP* and *pknB* is conserved in all Firmicutes and Actinobacteria (Fig. S1). PknB homologues, characterised by periplasmic PASTA domains (Penicillin-binding protein And Serine Threonine kinase Associated^20^), regulate many events in the cell cycle, as demonstrated by diverse phenotypes caused by gene disruption in *Bacillus subtilis*, *Staphylococcus aureus*, *Corynebacterium glutamicum*, *Streptomyces coelicolor*, *M. tuberculosis* and *Streptococcus pneumoniae*^21^. The mechanisms by which PknB regulates cell division are being elucidated and some may be organism-specific, for example PknB regulates peptidoglycan biosynthesis in *M. tuberculosis* by phosphorylation of several key enzymes and regulators including the regulatory protein CwlM^22,23^ and proposed lipid II flippase^18^.

PstP phosphatase function is also implicated in regulation of cell wall biosynthesis or cell division: a group B Streptococcus knockout had increased phosphorylation of cell division proteins^24^, *S. pneumoniae* knockout had defects in cell size and location of peptidoglycan synthesis^25^, *S. aureus* knockout had thickened cell wall^26^, and *C. glutamicum* knockout was slow-growing with pleiomorphic bulging cells. While this study was in progress, PstP was shown to be essential in *M. tuberculosis* and *Mycobacterium smemgatis* because depletion of PstP led to cell wall defects^27^. However, the cognate substrates of PstP and the mechanism by which it regulates cell cycle processes were unknown.

We hypothesised that PstP might play two roles: as a global negative regulator of protein S/T phosphorylation, and as a specific regulator of PknB and PknA by dephosphorylating enzymes and regulators of cell wall biosynthesis and cell division. We characterised *M. smegmatis* during PstP depletion, measuring changes in growth and viability, cell morphology and the phosphoproteome. These changes clarified the phosphoproteins regulated by PstP and support both roles.

## Results

### Knockdown of *pstP*-operon prevented growth of *M.smegmatis*

**Figure 2:**
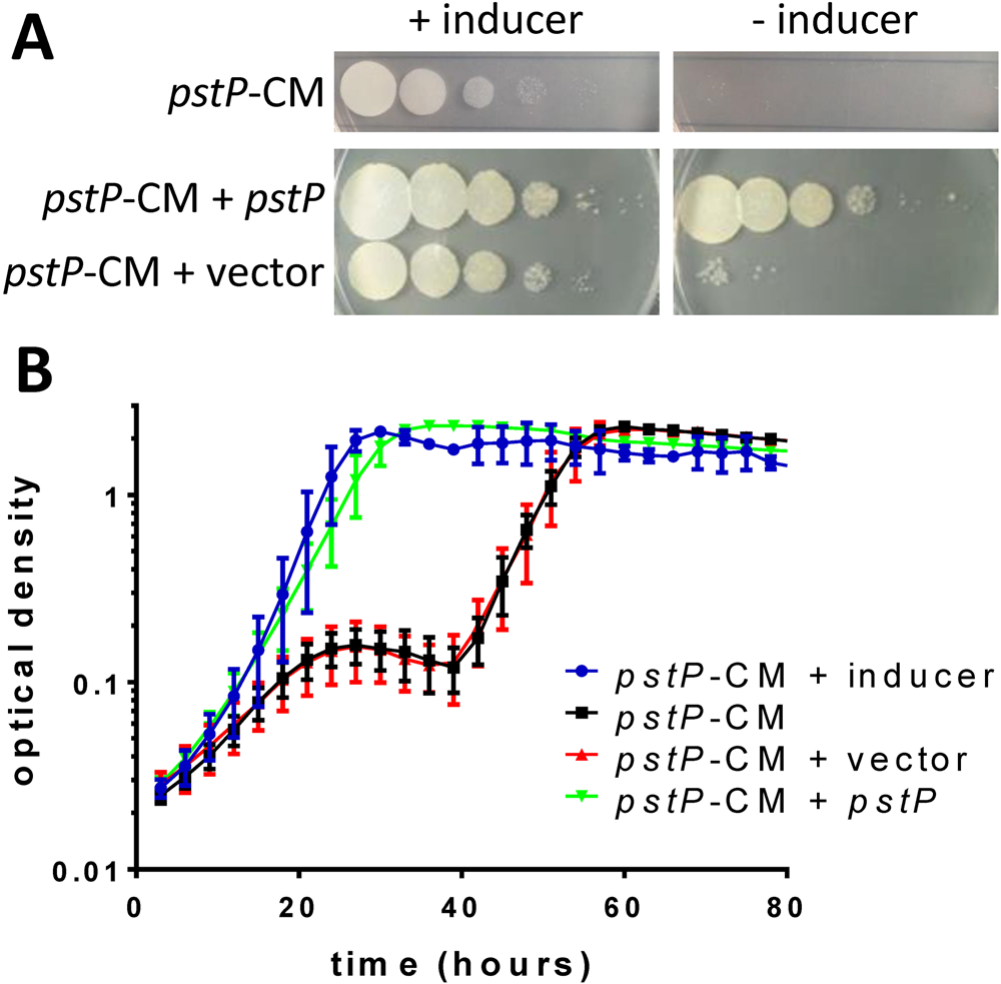
Knockdown of *pstP* prevented growth of *M. smegmatis*. Conditional mutant strain (*pstP*-CM), in which the *pstP* operon was under the control of the pristinamycin promoter, was only able to grow when the inducer pristinamycin was included in the medium. (**A**) Knockdown of the *pstP* operon by omission of inducer led to a 10^4^-fold drop in the number of colonies on 7H10 agar. Vector-borne *pstP*, but not control vector, restored the ability of the conditional mutant to form colonies in the absence of inducer. The three strains were cultured in liquid medium in the presence of inducer, washed, and serial 10-fold dilutions were spotted on plates with the appropriate antibiotics. (**B**) Knockdown of the *pstP* operon by removal of inducer led to cessation of growth in 7H9 broth, then a decline in optical density, followed by emergence of suppressor mutants. Vector-borne *pstP*, but not control vector, restored the ability of the conditional mutant to grow in the absence of inducer. Error bars show the standard deviation of four cultures. Graphs are representative of three independent experiments.

To gain insight into the cellular processes regulated by PstP, a conditional mutant was constructed. The *pstP* promoter was replaced with the pristinamycin inducible promoter (Fig. 1B) using the method of Forti *et al*.^28^. The resulting conditional mutant strain, *pstP*-CM, was expected to express *pstP* only in the presence of inducer. Based on the demonstration of *pstP* essentiality using the Tet-OFF system, *pstP*-CM was predicted to depend on inducer for growth on agar and liquid medium, as seen in Fig. 2A. Integration of a second copy of *pstP* at the *attP* site restored the ability to grow without inducer (Fig. 2A).

Knockdown of the *pstP* operon by removal of inducer led to cessation of growth in liquid medium, followed by a decline in optical density, suggestive of lysis (Fig. 2B). The low number of colony forming units after 22–32 hours of knockdown (Fig. S2) suggests loss of viability. After prolonged knockdown (40 hours, Fig. 2B) growth resumed, presumably due to the selection of second site mutations (suppressor mutations) since subsequent cultures without inducer lacked the prolonged lag phase.

Growth curves and light microscopy were used to identify the earliest time at which knockdown of the *pstP* operon led to changes in growth and morphology without loss of viability. During the first six hours, representing 2–3 generation times, growth appeared similar between induced and knockdown conditions (Fig. S3), but bulging cells began to appear (Fig. S4), which became more common after 24–30 hours of knockdown. These results led to the choice of six hours knockdown to characterise the proteomic responses to PstP depletion and 30 hours knockdown to characterise the changes in morphology.

### Withdrawal of inducer led to knockdown of *pstP* and *pbpA* expression

To characterise protein expression changes resulting from knockdown, the total proteome was compared after 6 hours culture with/without inducer (Fig. S5). 2794 proteins were detected, representing 42% of the proteome, which is similar coverage to previous proteomic characterisation of *M. smegmatis* (2550 proteins^29^). Only five proteins showed significant changes in abundance (Table 1). PstP and PbpA both decreased 5.7-fold (Table 1, Fig. S5), confirming successful knockdown and confirming co-transcription of these genes (and therefore presumably *rodA*, although RodA was not detected in the proteome in either condition, likely due to its multiple transmembrane helices). Protein kinases PknA and PknB decreased to a lesser extent, confirming the presence of additional promoter(s) downstream of *pstP* that contribute to their expression. The conditional mutant strain *pstP*-CM is therefore considered a triple knockdown of *pstP*, *rodA* and *pbpA*. Reintroduction of *pstP* restored growth (Fig. 2) despite knockdown of *rodA* and *pbpA*. This suggests that the essential kinases *pknA* and *pknB* were expressed independently from *pstP*. The soluble phosphatase MspP was detected in the proteome, though less abundant than PstP, and its abundance did not change during knockdown conditions.

**Table 1:** Comparative proteomics identified five proteins that were significantly less abundant (>2 fold change, p < 0.05) after conditional knockdown of the *pstP* operon.

Protein name/function	Gene number MSMEG_	Decrease in abundance (fold change)
Ribosomal protein L35	3792	6.55
PstP protein phosphatase	0033	5.76
PbpA penicillin-binding protein A	0031	5.72
Conserved transmembrane protein	6099	3.87
Short-chain dehydrogenase/reductase	3302	2.49

The lack of change in global protein levels after 6-hours knockdown (Fig. S5) suggests that this time point is appropriate to investigate changes in protein phosphorylation in response to PstP depletion before widespread changes in gene expression.

### *pstP*-operon knockdown led to changes in the cell wall appearance

**Figure 3:**
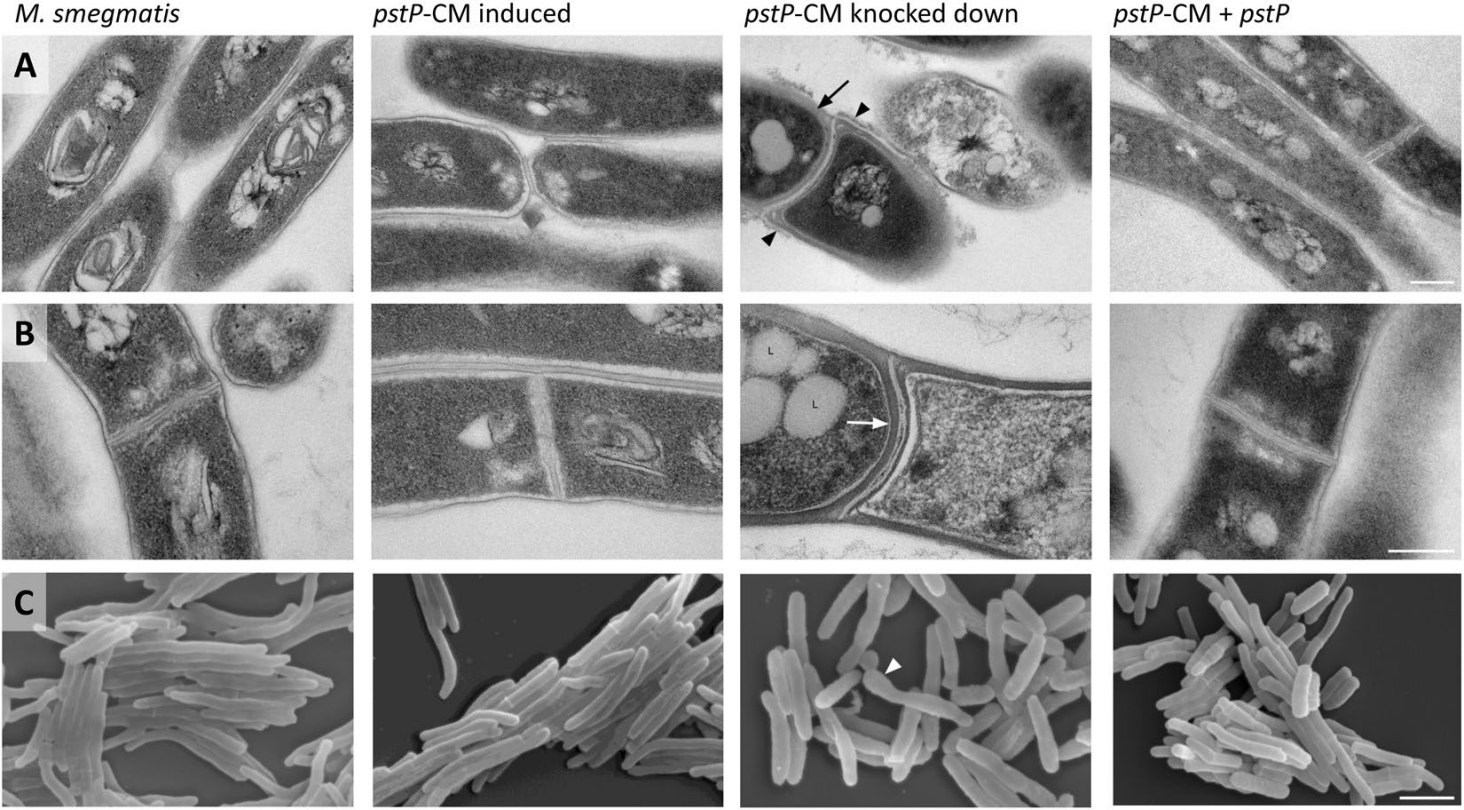
Knockdown of the *pstP* operon led to changes in the cell wall and cell shape. Knockdown for 30 hours led to changes in *pstP*-CM compared to wild type *M. smegmatis*, and some of these changes were reversed in the complemented strain *pstP*-CM + *pstP*. Cells were imaged by transmission electron microscopy (**A**,**B**) and scanning electron microscopy (**C**). PstP knockdown led to thicker cell wall (black arrow), thicker septum (white arrow), and bulging cells with lysis (white arrowhead). These defects were complemented by reintroduction of *pstP*. Knockdown of the *pstP* operon also led to rough cell surface (black arrowhead) and lipid bodies (L) that were partially complemented by reintroduction of *pstP*, and reduced cell length (quantified in Fig. S8) that was not complemented by reintroduction of *pstP*. Images within a single row are shown at the same scale, which is indicated by a bar in the right hand image: 200 nm for TEM (**A**,**B**) and 2.5 μm for SEM (**C**). Images are representative of at least twelve fields of view: additional fields for transmission electron microscopy are presented in Figs S6 and S7, and cell lengths for 100 cells are presented in Fig. S8.

Transmission electron microscopy was used to characterise the effects of knockdown of the *pstP* operon on cell morphology, revealing a thickening of the cell wall, septal defects, rougher cell surface and accumulation of intracellular lipid bodies (Figs 3A,B, S6 and S7). Scanning electron microscopy revealed a reduction in cell length as well as bulging cells and evidence of lysis (Fig. 3C). Reintroduction of *pstP* restored the cell wall thickness and morphology of the septa (Figs 3A,B, S6 and S7), suggesting that these defects were due to loss of *pstP*, and that the regulatory role(s) of PstP might encompass cell wall synthesis or cell division. Other aspects of the phenotype were partially complemented (surface roughness and lipid bodies, Figs 3A,B, S6 and S7) or not complemented (cell length, Figs 3C and S8), indicating the influence of knockdown of other genes in the *pstP* gene cluster in the control of these features. Indeed, knockdown of *rodA* or *pbpA* have each been found to perturb cell length and cell wall thickness in *M. tuberculosis*^12^, while Tet-OFF knockdown of *pstP* resulted in elongation of *M. tuberculosis* and a multiseptum phenotype in *M. smegmatis*^27^. Multiseptate cells were not abundant after *pstP-*operon knockdown, highlighting the interdependence of gene function within this cluster, which means that conditional mutants constructed by different methods were not equivalent.

### *pstP*-operon knockdown led to global increases in protein phosphorylation

**Figure 4:**
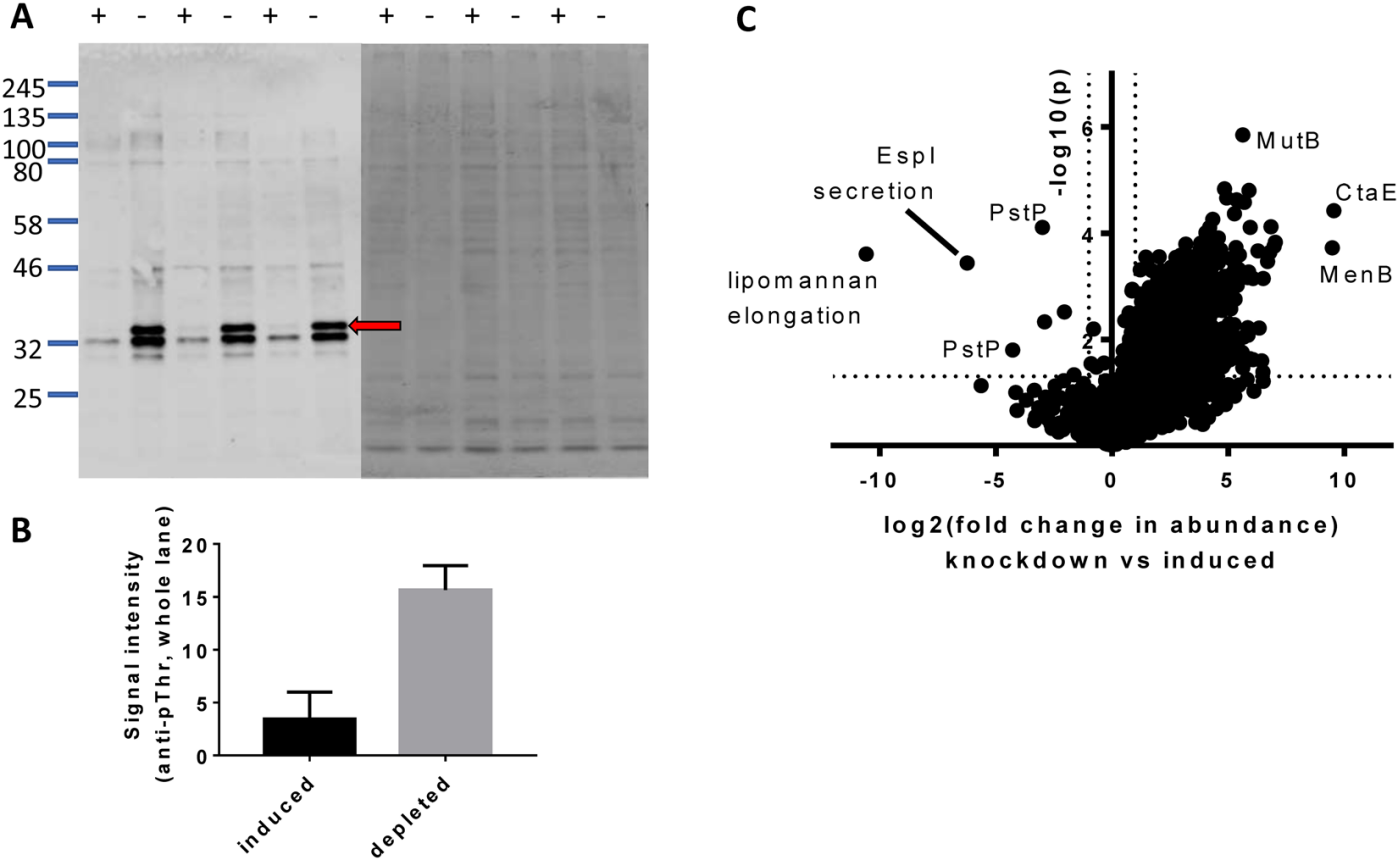
PstP knockdown led to increased protein phosphorylation. (**A**) Removal of inducer led to a global increase in the abundance of protein threonine phosphorylation. Lysates from *pstP*-CM grown for 6 hours in the presence (+) or absence (−) of inducer were normalized for protein concentration by Coomassie staining (right), blotted and probed with anti-phosphothreonine antibody (left). The increase in protein threonine phosphorylation was non-uniform: a differentially enriched phosphoprotein is marked with a red arrow. (**B**) The increase in signal upon anti-pThr probing was significant (densitometry and students t test, p < 0.05; data are the mean and standard deviation of 3 independent replicates). (**C**) Comparative phosphoproteomics identified phosphosites that changed significantly upon knockdown. Dashed lines indicate thresholds for significance: >2 fold-change and p < 0.05.

Knockdown of PstP was expected to increase the level of phosphorylation of PstP substrates. Increased protein threonine phosphorylation during knockdown was detected using a phosphothreonine specific antibody (Fig. 4A), confirming that PstP negatively regulates protein phosphorylation on threonine in *M. smegmatis*. A phosphoprotein of approximately 40 kDa was notable for its abundance and striking increase in phosphorylation during PstP depletion (Fig. 4A). Based on the abundance, molecular weight and fold-change in the phosphoproteome, this protein is tentatively identified as the menaquinone biosynthetic enzyme MenB (Supplementary Table S1).

Comparative phosphoproteomics allowed identification of differentially phosphorylated proteins during knockdown of the *pstP* operon. We identified 1693 phosphosites on 894 proteins in total. Almost half the phosphosites (838) were significantly more abundant in the knockdown condition (Fig. 4B and supplementary files). The wide-scale increase in phosphopeptide abundance (Fig. 4) without large changes in protein expression (Fig. S5) confirm the role of PstP as a global negative regulator of S/T phosphorylation in *M. smegmatis*.

**Figure 5:**
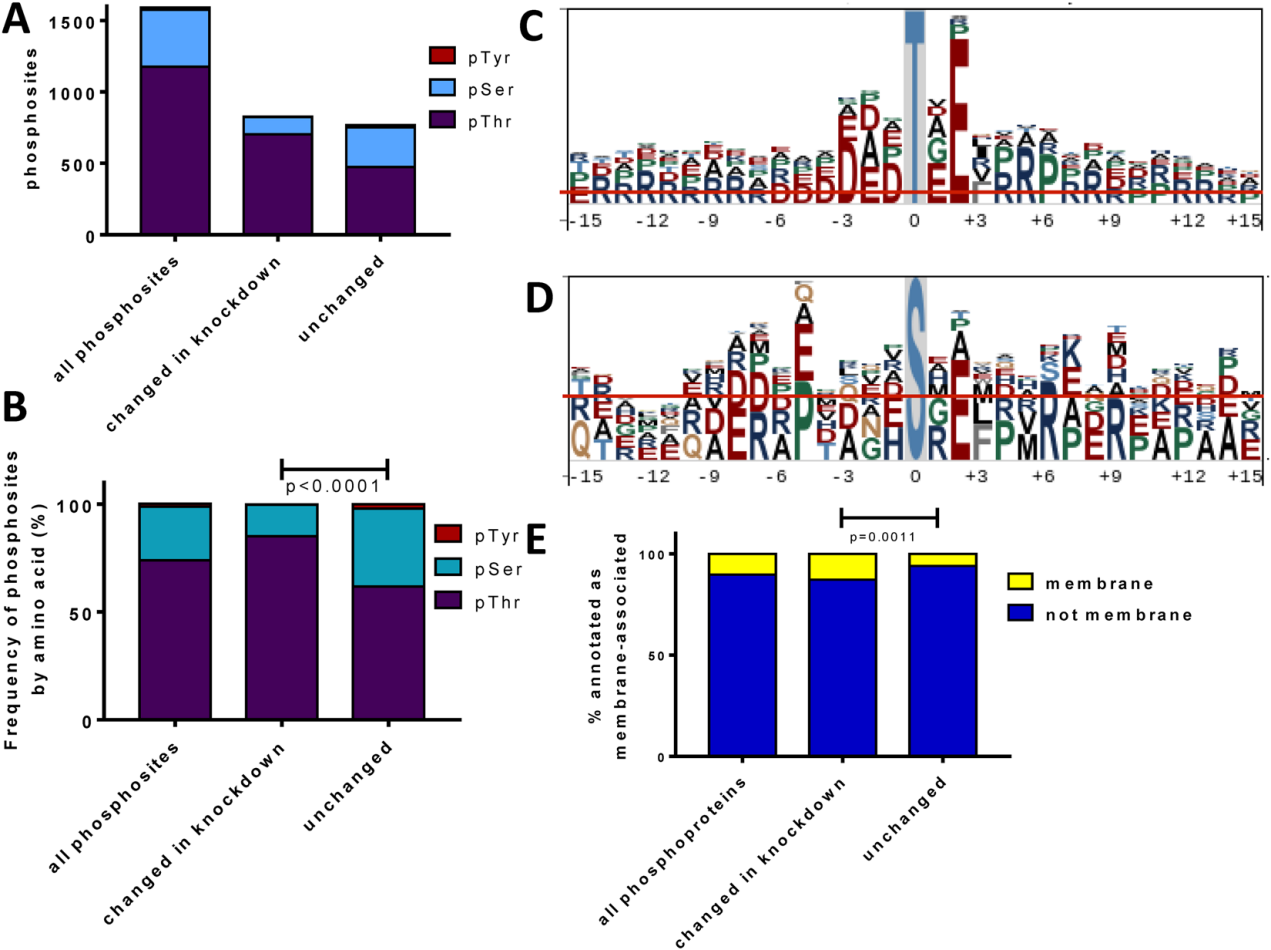
Sequence and functional analysis of phosphosites. (**A**) Threonine was the most frequent site of phosphorylation. (**B**) Knockdown of the *pstP* operon led to a differential increase in phosphorylation at pThr. (**C**,**D**) Sequence motifs surrounding pThr and pSer sites with highest increase during knockdown (>8-fold change). (**E**) *pstP* knockdown led to a differential increase in phosphorylation on proteins annotated as transmembrane or membrane associated.

Profiling the phosphopeptides that changed upon PstP knockdown could clarify the functional classes of proteins that are regulated by PstP and give an indication of the primary sequence motifs of phosphosites that are regulated by PstP. Phosphothreonine (pT) was the most abundant phosphosite (Fig. 5A), as seen in Mycobacteria and other bacteria^30^. Furthermore, pT was significantly enriched in the phosphosites that changed in response to knockdown (Fig. 5A), fitting with the observed *in vitro* activity of *M. tuberculosis* PstP towards pT and pS^3^.

Motif analysis of the global phosphoproteome revealed a preference for acidic residues on either side of the phosphorylation site (Fig. 5B), which is similar to the *in vitro* specificity of *M. tuberculosis* protein kinases^31^. Furthermore, amongst the phosphosites changed during knockdown there was a bias towards phosphorylation sites near the N-terminus (10% of the changed phosphopeptides were phosphorylated at position two, and 20% within the first 12 residues, a significant difference compared to the positions of unchanged phosphosites). The greater frequency and more labile nature of phosphorylation near the protein termini is expected from the more exposed nature of the termini compared to residues in defined protein folds^12^, making them more accessible to kinases and phosphatases.

Protein localisation is also likely to influence substrate access to PstP. Membrane proteins and membrane associated proteins were significantly enriched amongst the differentially phosphorylated proteins, fitting with the membrane localisation of PstP (Fig. 5C).

The functional classes of proteins that are differentially phosphorylated could reveal specific cellular processes that are regulated by PstP. However, half of all phosphopeptides were increased upon knockdown, representing all functional classes (supporting information). The ten phosphoproteins with the greatest increase in abundance (Table 2) include enzymes involved in vitamin biosynthesis, central carbon metabolism and transcription.

**Table 2:** Comparative phosphoproteomics identified phosphosites that were more abundant upon *pstP* knockdown.

Protein name/function	Gene name MSMEG	Peptide (phosphosite in bold)	p-site	Fold change
Cytochrome c oxidase subunit 3 CtaE	4260	T**S**AVGTSGTAITISR	S3	750
Menaquinone biosynthesis MenB	1075	TGYQYASGE**T**AETVDPAR	T117	720
Transaminase (biotin metabolism)	2450	**T**AIQESTLLPNGLTVDTAK	T2	131
PEP carboxylase Ppc	3097	ADSND**T**ALEPFGSVQR	T7	126
isopentenyl diphosphate synthesis IspG	2580	T**S**IGLGMPAPPAPTLAPR	S3	114
Heparin binding heamagglutinin-like	0919	VETYTDQAVEL**T**QEALGTVASQTR	T144	111
RNA polymerase RpoC	1368	NIQVQP**T**EEAR	T1276	104
Conserved hypothetical	3032	HGLAADDDRAD**T**DVFAAVSANGAQEHTDTTQTTPLENPDQPR	T159	92
ATP synthase epsilon chain AtpC	4935	IAVDGGFLSV**T**EETVR	T73	91
Uncharacterised	6336	**T**SDLPASPDQPEPTHGMPPPPPAPGKPK	T2	90
methyltransferase	1049	**T**EIRDAADPAPNPHATAEEVEAAMHDSK	T2	87
transketolase	3103	LVGDTGEIVSIEHYGESADDK**T**LFR	T674	81

The phosphosites with greatest fold-increase (p < 0.05) are listed and the full dataset is available as supporting information.

**Figure 6:**
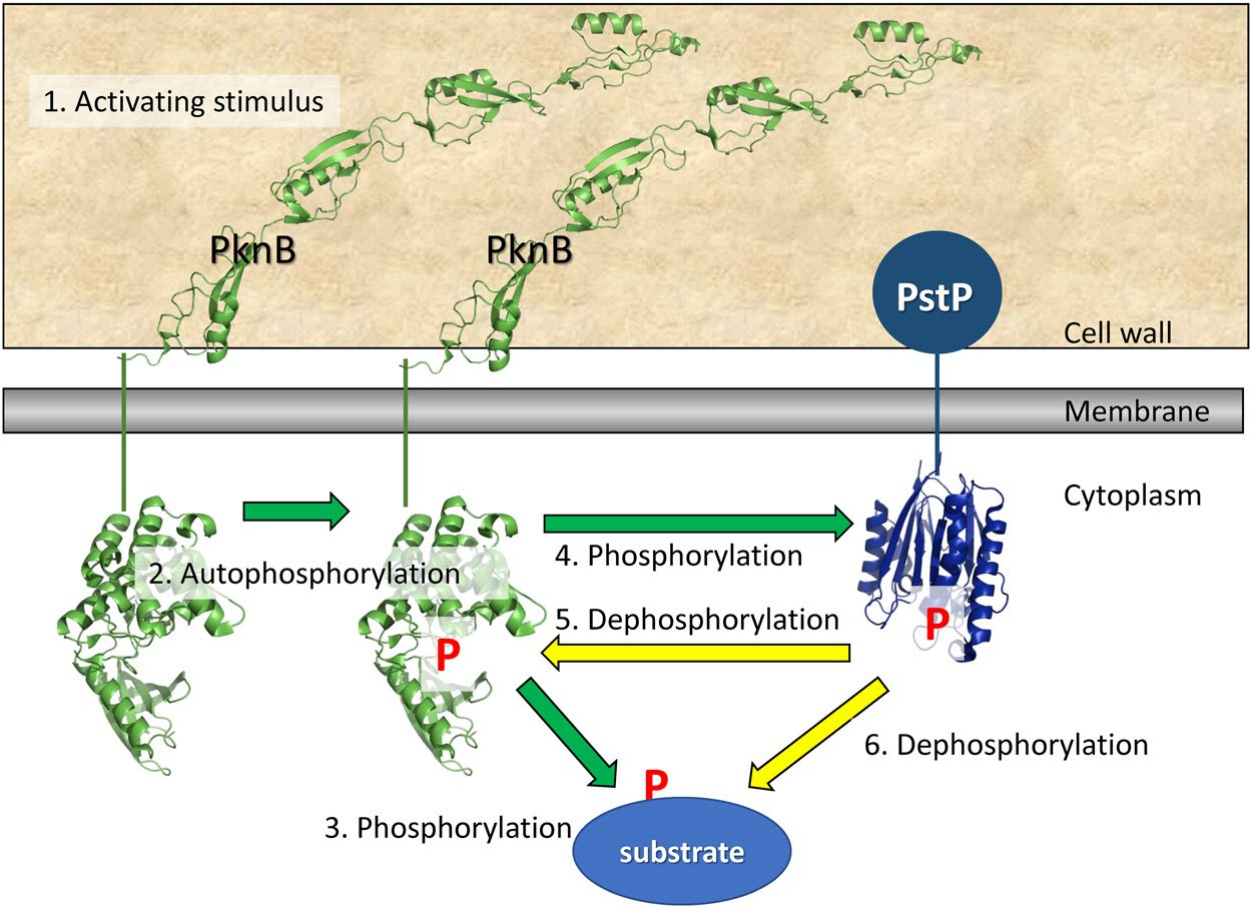
Negative feedback between PknB and PstP allow a transient response to stimulation. 1 Transient stimulation of PknB. 2 PknB autophosphorylates becoming activated. 3 Activated PknB phosphorylates its substrates. 4 Activated PknB phosphorylates PstP, activating PstP. 5 Activated PstP dephosphorylates PknB, returning it to the unactivated form. 6 Activated PstP dephosphorylates PknB substrates (including itself). PknB and PstP oppose each other, so that the response to PknB stimulation is transient. When PstP is knocked down there is no negative regulator to counter the positive feedback of PknB autoactivation, so transient stimuli lead to lasting PknB activation and widespread hyperphosphorylation of PknB substrates. Structures deposited in the PDB are used in this scheme (1O6Y, 2KUI, 2CM1), although the structural changes that occur during signalling are unknown. Similar feedback loops may exist for other Mycobacterial protein kinases.

PstP knockdown has direct and indirect routes to increase protein phosphorylation (Fig. S9). Notably, PstP curtails or reverses PknB activation by dephosphorylating the activation loop^3,32^, and this is likely the case for other kinases^33^. Conversely, PknB and PknA phosphorylate PstP to increase its activity^13^, suggesting a possible homeostatic mechanism (Fig. 6). It was therefore important to determine which of the 18 *M. smegmatis* S/T protein kinases became activated during PstP knockdown, since kinase activation would influence the phosphoproteome. Phosphorylation sites were detected in seven of the kinases (Table 3). Fourteen phosphosites were significantly more abundant in the knockdown condition, while only one site on PknG was significantly less abundant. Phosphopeptides from PknB and PknD had the greatest fold change, notably including the active site loop of PknB (Table 3), thus the changes in the phosphoproteome reflect both decreased PstP activity and the ensuing increase in activity of PknB and other kinases.

**Table 3:** Phosphorylation sites on Ser/Thr protein kinases that were significantly changed during *pstP* knockdown.

Kinase	Peptide (phosphosite in bold)	p-site	Fold change
PknB MSMEG_0028 A0QNG1	ALAD**T**GNSVTQTAAVIGTAQYLSPEQAR	T166	49.5
	ALADTGNSVTQTAAVIG**T**AQYLSPEQAR	T179*	36.1
	AGAA**T**QDMPVPRPAGYSK	T309	5.8
	VL**T**DAER	T289	5.5
PknD MSMEG_4366 A0R0F3	R**T**PTPPAPAAVAAPAVR	T333	32.0
	TP**T**PPAPAAVAAPAVR	T335	6.9
	QFSELYPNPEHTGFTP**Y**PPAYPSVPAPPR	*Y319*	*−2.1*
PknG MSMEG_0786 A0QQK3	GTVVTEAYDQVTMA**T**R	T75	18.5
	PMATQAVYRPEFDD**T**DGTSR	T55	−3.1
PknA MSMEG_0027 A0QNG2	RPPRPNQAP**T**LGR	T288	4.2
	RPFTGDGAL**T**VAMK	T224	2.5
	DVKPGNILITP**T**GQVK	T152	2.4
	RPPRPNQAP**T**LGR	T288	4.2
PknF MSMEG_3677 A0QYJ2	LGTDADVADPDA**T**R	T290	3.2
	SPFHH**S**NAAVVISK	*S221*	*3.0*
PknL MSMEG_4243 A0R032	FHNRPQPDLE**T**TAEVAPPPPAPATVPR	T316	2.3
	FHNRPQPDLET**T**AEVAPPPPAPATVPR	T317	2.3

* indicates phosphosites in the kinase activation loop.

To test whether higher PknB activity could account for some of the increased phosphorylation seen during knockdown of the *pstP* operon, we searched our phosphoproteome dataset for substrates of PknB identified in previous proteome-wide studies^22,23,34^. Phosphosites in CwlM, MurJ (MviN), the transporter MSMEG_1642 (Rv1747-homologue), FabG, SigH and EspI were increased >16-fold during knockdown. CwlM, MviN and Rv1747 are involved in synthesis, regulation and export of cell wall components. Phosphorylation-dependent changes in their activity could contribute to the cell wall thickening seen by electron microscopy (Fig. 3A). Thus prolonged activation of PknB is a plausible contributor to the increased phosphorylation during knockdown of the *pstP* operon. However, the changes in phosphorylation are likely to result from a combination of factors, especially taking into consideration that additional proteins that are not known substrates of PknB were also hyperphosphorylated in the knockdown condition.

Only seven phosphopeptides were significantly decreased in the knockdown condition (Table 4), and these derived from proteins involved in cell wall synthesis and transmembrane transport. The greatest decrease in phosphorylation was at T369 of MSMEG_0317, which is a transmembrane protein involved in lipomannan maturation and lipoarabinomannan synthesis in *C. glutamicum* (NCgl2760) and which is essential in both *M. tuberculosis* (Rv0227) and *M. smegmatis*^35^. It is tempting to speculate that this reflects a role for PstP in control of cell wall processes, fitting with the changes in morphology observed during prolonged PstP knockdown (Fig. 3). PstP itself was one of the  proteins with decreased phosphorylation in the knockdown sample. The change in phosphopeptide abundance (19.3- and 8.0- fold) exceeded the change in protein abundance (5.7-fold), providing support for previous findings that PstP may itself be regulated by phosphorylation^13^.

**Table 4:** Phosphosites that decreased in abundance upon *pstP* knockdown (threshold >2-fold change; p < 0.05).

Protein name/function	Gene name MSMEG_	Peptide (phosphosite in bold)	p-site	Fold change
Lipomannan elongation	0317	TESTLIDPGLDTADHGFFD**T**QGIQVPGAEAK	T369	1534
EspI regulator of ESX secretion	0067	LFH**S**SEPGKPVDDATITVDR	S11	74.9
PstP	0033	I**T**AEEAHSHPQR	T146	19.3
PstP	0033	ITAEEAH**S**HPQR	S152	8.0
Peptidoglycan binding LysM	5526	VIEAPELDDLND**T**DDLEPVRLDIPSEFLADAR	T52	7.5
PEP protein phosphotransferase PtsI	0088	TLDAG**S**DKPLK	S322	4.1
PknG	0786	PMATQAVYRPEFDD**T**DGTSR	T55	3.1

## Discussion

PstP is required for normal cell wall synthesis and cell division, as demonstrated by the cell wall and morphological changes with loss of viability upon knockdown of the *pstP* operon. Differential phosphorylation of cell division-related proteins during PstP knockdown revealed possible mechanisms by which PstP might regulate peptidoglycan synthesis by dephoshorylating PknB and CwlM, as well as other cell cycle related proteins including Rv0227, MurJ (MviN) and FhaA. Further work will be required to characterise the specific effects of PstP dephosphorylation on the activity of each. The soluble S/T phosphatase MspP, which shares 38% amino acid identity with the catalytic domain of PstP, remained undisrupted in the knockdown (*mspP* is in a distant genetic locus and there was no significant change in protein abundance) thus MspP cannot substitute for the function of PstP. No MspP phosphosites were identified during phosphoproteome analysis.

Western blot and phosphoproteomics suggest additional, broader roles for PstP. The changes in phosphorylation upon knockdown of the *pstP* operon surpassed those observed in previous phosphoproteomic comparisons of gene disrupted bacteria in the number and proportion of proteins that were differentially phosphorylated and in the magnitude of the fold-change in the phosphopeptides. The outnumbering of PstP by 18 S/T kinases implies that PstP depletion could affect the concentration of many phosphoproteins but the high fold-change values was unexpected given the mild and brief period of PstP depletion: by 6 hours PstP concentration fell 5.7-fold while 52 phosphopeptides increased >32-fold and globally 50% of phosphosites were significantly changed with 22% increased by over 8-fold. By comparison, knockout of S/T kinases or phosphatase in other bacteria led to fewer significant changes and lower fold-change: 8 out of 64 sites in *B. subtilis* S/T phosphatase knockout, 55/603 in *Mycobacterium bovis pknG* knockout^36^ and 19/456 in *M. tuberculosis pknB* knockdown^23^. A possible explanation for the large, extensive and rapid changes in phosphorylation emerged from examining differential phosphorylation of protein kinases. PknB had the greatest increase in phosphorylation, including at threonines in and near the activation loop (49-fold increase in pT166 and 36-fold increase in pT179). *In vitro* evidence suggests that PknB and PstP regulate each other^3,8^. Loss of PstP would lead to prolonged PknB activation^32^, and potentially to positive feedback from uncontrolled autoactivation of PknB (Fig. 6). The dramatic and extensive changes in the phosphoproteome that arise from PstP disruption would fit with this model. Thus, differential phosphorylation in the proteome during PstP knockdown could result from a combination of prolonged PknB activation causing increased phosphorylation of PknB substrates, as well as slower dephosphorylation of PstP substrates. The observed substrate preferences (Fig. 5) may reflect a combination of the preferences of PknB and PstP. PknB was the only kinase whose activation loop was detected in the phosphoproteome, but we cannot exclude that PstP normally curtails activation of other kinases. Remembering that knockdown of the *pstP* operon is eventually lethal, it is likely that the massive up-regulation of phosphoproteins is due to non-physiological conditions.

The phosphoproteomic and genetic evidence linking the function of PstP and PknB provides a mechanism by which PstP can regulate cell wall synthesis by dephosphorylation of the substrates of PknB (and PknA) and by dephosphorylation of PknB itself to reduce activation. Supporting this explanation are similarities between the morphological defects that result from PstP-depletion and PknB overexpression: both result in widened, bulging, pleiomorphic cells (Fig. 3 and^17,27^) albeit with possible confounding effects from the growth medium, growth phase and polar effects^12^. Similarly PstP overexpression and PknB depletion both result in longer cells^23,27^. Overall, the non-uniform increase in protein phosphorylation upon knockdown of the *pstP* operon demonstrates that the role of PstP is more complex than general dampening or reversal of the effects of protein kinases. Phosphorylation changes upon knockdown were enhanced at certain motifs and on certain substrates. A study of the PstP homologue in *Enterococcus faecium* also found evidence of substrate selectivity when recombinant phosphatase was used to dephosphorylate whole cell extracts^9^.

In summary, PstP is a regulator of cell wall synthesis and cell division and is also a global regulator of protein S/T phosphorylation thus influencing all cellular processes. The first role in cell division may occur directly by the action of PstP of the enzymes and regulators of cell wall synthesis or indirectly by specifically controlling the activity of PknB, or by both mechanisms. The general role in global regulation may also occur directly through substrate dephosphorylation, or indirectly by specific dephosphorylation of the activation loops of kinases, or by both routes.

## Methods

### Bacterial strains and culture conditions

*M. smegmatis* mc^2^155 was routinely cultured at 37 °C on Middlebrook 7H9 (Becton, Dickinson and Company) liquid media supplemented with 10% (v/v) Albumin-Dextrose Complex (0.5% bovine serum albumin, 0.2% dextrose, 0.085% NaCl), 0.2% (v/v) glycerol and Tween 80, 0.05% (w/v). Antibiotics were used as follows: the conditional mutant *pstP*-CM was routinely cultured in the presence of hygromycin (50 μg/ml) and pristinamycin (2 μg/ml), and *pstP*-CM transformed with pMV306 or derivatives was routinely cultured in the presence of hygromycin, pristinamycin and kanamycin (30 μg/ml).

### Gene cloning

*pstP* with the cognate promoter was PCR amplified from *M. smegmatis* genomic DNA and cloned in the KpnI and HindIII sites of pMV306^37^. [aaaaGGTACCggtgacaagcagcccgcttcac, aaaaAAGCTtcatgacaccgcccggcag]

### Generation of conditional knockout and complemented strains

The 5′ end of *pstP* was PCR including the ribosome-binding site (402 bp) was PCR amplified from *M. smegmatis* genomic DNA [actCCATGGaggagcagaatgaccctcgttct, AcaGCATGCttggtgatctgtgtcagctcacc] and cloned under the control of the pristinamycin inducible promoter in the NcoI and SpeI sites of pAZI9479^28^ in order to disrupt the genomic *pstP* locus by homologous recombination. The sequenced plasmid was transformed into *M. smegmatis* by electroporation and selection with hygromycin. The site of recombination was confirmed by PCR [aggagcagaatgaccctcgttct, acacgttcgtgcagaccctcgt].

### Determining the effects of knockdown on growth on solid and liquid medium

*M. smegmatis pstP*-CM was cultured in Middlebrook 7H9 broth with appropriate antibiotics (hygromycin for *pstP*-CM; hygromycin and kanamycin for *pstP*-CM transformed with pMV306 or pMV306-*pstP*). Cells were washed in 7H9 without inducer, serially diluted, and spotted in parallel onto 7H10 plates with/without inducer. Plates were incubated at 37 °C for three days and used to take photographic images. Images are representative of 3 independent experiments.

To determine the effects of knockdown of the *pstP* operon on growth in liquid medium, cultures were prepared and washed as above, and used to inoculate parallel cultures with/without inducer. The initial optical density was 0.05. Cultures were grown at 37 °C with shaking in a BioScreen (Fig. 2) or conical flasks (supplementary figures), and optical density was monitored.

### Transmission Electron Microscopy (TEM)

*M. smegmatis* strains were seeded at OD 0.001 in 7H9 with/without inducer and incubated at 37 °C with shaking for 30 hours. Bacterial cells were harvested and washed in PBS then fixed in 2.5% glutaraldehyde in PBS. An aliquot was saved for scanning electron microscopy (see below) and the remainder were pelleted in 3% agar, cooled, and cut into 1 mm^3^ cubes. Agar cubes were treated with 1% w/v aqueous osmium tetroxide, dehydrated through an ethanol series, and embedded in Spurr’s Resin. Samples were sectioned to a thickness of approximately 70 nm using a Reichert Ultracut E ultramicrotome and collected onto copper mesh grids. Dry grids were submerged briefly in methanol before staining in 2% (w/v) uranyl acetate for 30 minutes followed by Reynolds lead citrate for 5 minutes. Samples were viewed on a JEOL JEM-1400 TEM with an accelerating voltage of 100 kV. Images were collected using a Megaview III digital camera with iTEM software.

### Scanning Electron Microscopy (SEM)

An aliquot of glutaraldehyde-fixed cells (10 µl of each strain) was applied to a Poly-L-lysine treated glass slide, then further treated with 0.5% (w/v) aqueous osmium tetroxide and dehydrated through ethanol and anhydrous hexamethyldisilazane. Dried slides were mounted onto aluminium stubs and sputtercoated with gold/palladium in a Quorum Q150TES coating unit. Bacteria were viewed on a Hitachi S3000H SEM with an accelerating voltage of 10 kV. The length of 100 cells of each strain was measured using ImageJ and differences between the strains were analysed by Mann Whitney test.

### Preparation of cell extract for proteomic and Western blot analysis

Exponential phase *pstP-*CM was washed and used to seed parallel 200 ml cultures with and without inducer at initial optical density of 0.1. Cultures were incubated at 37 °C with shaking and harvested after 6 hours. Replicate cultures were used for Western blotting and for proteomic analysis. Cells were resuspended in extraction buffer (ammonium bicarbonate 100 mM, NaCl 1 M, Urea 8 M, DTT 20 mM, PhosSTOP phosphatase inhibitors and cOmplete protease inhibitors (Roche)). Cells were disrupted using a Minilys personal homogenizer (Bertin instruments) using glass beads (Sigma) and cell debris was removed by centrifugation at 20,000 g for 30 minutes. The supernatant was concentrated using 1000 kDa molecular weight cut-off filters and protein concentration estimated using Qubit assay (Thermofisher). Protein concentration was normalized before Western blot analysis, and normalization was verified by parallel SDS PAGE with Coomassie blue staining. Polyclonal phosphothreonine antibody was purchased from Cell Signalling and used according to the manufacturer’s instructions.

### Comparative proteomics and phosphoproteomics

*M. smegmatis pstP-*CM cell extracts were reduced and alkylated in extraction buffer containing 5 mM Tris(2-carboxyethyl) phosphine hydrochloride (TCEP) and 10 mM chloroacetamide (CAA), for 30 min at room temperature. Proteins were precipitated using the methanol-chloroform method^38^ then dissolved in 20 mM HEPES-Trypsin-LysC (1:100) and digested for 16–17hrs.

The tryptic peptides concentration was determined using Qubit. Part of the peptide sample (95%) was used for phosphoproteomics analysis and the remainder (5%) for proteome analysis. The peptides were dried using a SpeedVac concentrator and then resuspended in 80% acetonitrile, 5% trifluoroacetic acid (TFA). For the phosphoproteomics analysis, phosphopeptides were enriched using Titansphere (10 µm or 5 µm, 500 mg; 5020-75010 GL Sciences Inc. Japan) according to the manufacturer’s instructions. Dried peptides were resuspended in a buffer containing 2% acetonitrile and 0.1% TFA prior to analysis by mass spectrometry.

Reversed phase chromatography used an Acclaim PepMap µ-precolumn cartridge 300 µm i.d. × 5 mm, 5 μm, 100 Å and an Acclaim PepMap RSLC 75 µm i.d. × 50 *pstP*-CM, 2 µm, 100 Å (Thermo Scientific) using 0.1% formic acid and acetonitrile containing 0.1% formic acid. Eluted peptides were converted to gas-phase ions by means of electrospray ionization and analysed on a Thermo Orbitrap Fusion (Thermo Scientific).

MS raw files were analyzed by MaxQuant software version 1.5.5.3^39^ and peptide lists were searched by the Andromeda Search Engine^40^ against the *M. smegmatis* Uniprot database (Proteome IF UP000000757, 6601 proteins). Bioinformatics and statistical analyses were performed in the Perseus software (version 1.5.5.3). Confidently assigned phosphorylation sites with localization probabilities greater than 75% and Score difference greater than 5 were defined as Class I sites and were used as site localization cutoff from the Phospho (STY)Sites table generated by MaxQuant analysis. Missing data points were imputed by creating a normal distribution with a width of 0.3 and a downshift of 1.8. Student’s T-tests for statistical significance were performed with permutation-based FDR correction threshold of 0.05. PRIDE accession number: PXD011805.

## Supplementary information

Supplementary figures S1-S9

Table S1

## Data Availability

The datasets generated during the current study are available in PRIDE, accession number: PXD011805. https://www.ebi.ac.uk/pride/archive/login.
