# Supplementary material for: Mycobacterial phosphatase PstP regulates global serine threonine phosphorylation and cell division
Source: Sci Rep. 2019 Jun 6;9:8337. doi: 10.1038/s41598-019-44841-9 (PMC6554272; doi:10.1038/s41598-019-44841-9)
Supplement: Supplementary file 1 — Supplementary figures S1-S9 [file 41598_2019_44841_MOESM1_ESM.pdf]

Mycobacterial phosphatase PstP regulates global serine threonine phosphorylation and cell division

Iswahyudi, Galina V. Mukamolova, Anna A. Straatman-Iwanowska, Natalie Allcock, Paul Ajuh, Obolbek Turapov, Helen M. O'Hare

List of material

Figure S1. *pstP* and PASTA kinase are conserved in all Firmicutes, and the *pknB-fhaA* gene cluster is conserved in a minority of Firmicutes.

Figures S2. Knockdown of the *pstP* operon led to a decline in colony forming units

Figure S3. During the first 6 hours of knockdown there were no significant changes in bacterial growth rate determined by optical density.

Figure S4. Knockdown of the *pstP* operon led to bulging cells (indicated by red arrowheads).

Figure S5. PstP and PbpA were depleted after six hours of knockdown of the *pstP* operon, but without global changes in protein concentration.

Figure S6. Transmission electron microscopy showing the presence/absence of lipid bodies, and smooth/rough cell surface (additional fields, to accompany Fig 3).

Figure S7. Transmission electron microscopy showing cell wall and septum (additional fields, to accompany Fig 3).

Figure S8. Cell length was reduced during knockdown of the *pstP* operon, and the length was not restored by reintroduction of *pstP*.

Figure S9. Direct and indirect mechanisms by which knockdown of the *pstP* operon may influence protein phosphorylation and cell physiology.

Table S1 Comparative phosphoproteome of *pstP*-CM cultured in knockdown and induced conditions (excel spreadsheet)

Deposited data: raw and analysed phosphoproteomics data, ProteomeXchange Consortium via the PRIDE. Project accession: PXD011805

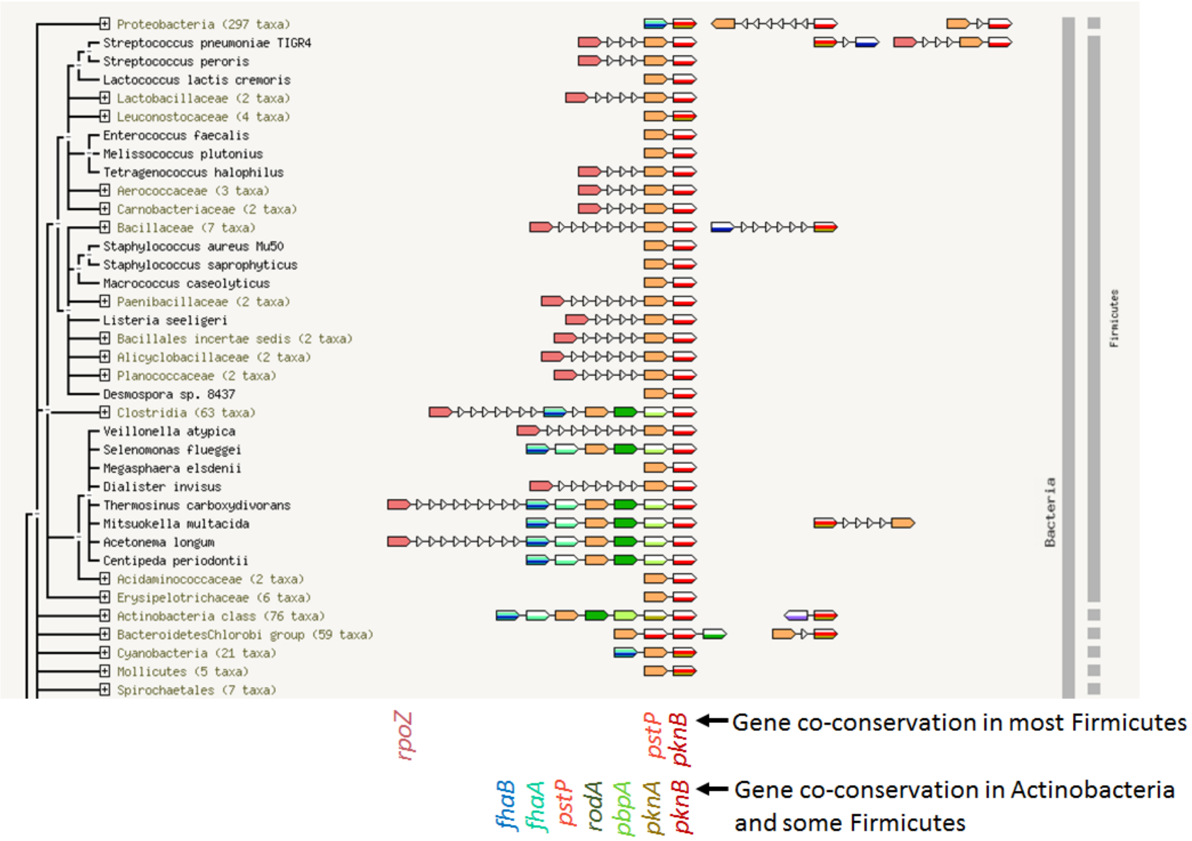

Figure S1. *pstP* and PASTA kinase are conserved in all Firmicutes, and the *pknB*-*fhaA* gene cluster is conserved in a minority of Firmicutes.

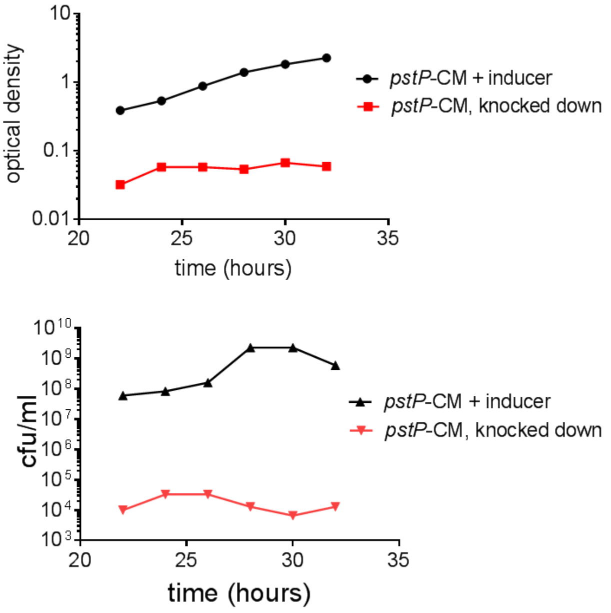

Figure S2. Knockdown of the *pstP* operon led to a decline in colony forming units. Knockdown for 20-30 hours led to a decline in optical density (Figure 1). To determine whether the decline in optical density was accompanied by a reduction in colony forming units, cultures were spotted onto Middlebrook 7H10 with pristinamycin. Results are representative of three independent flasks.

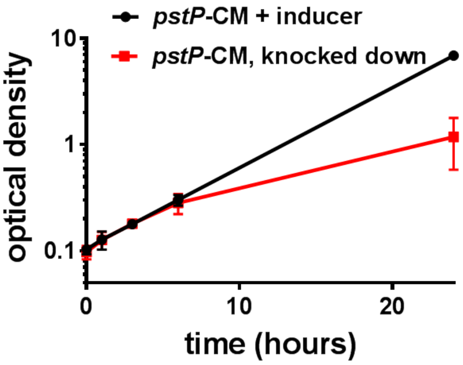

Figure S3. During the first six hours of knockdown there were no significant changes in bacterial growth determined by optical density. Results show the mean and standard deviation of three flasks.

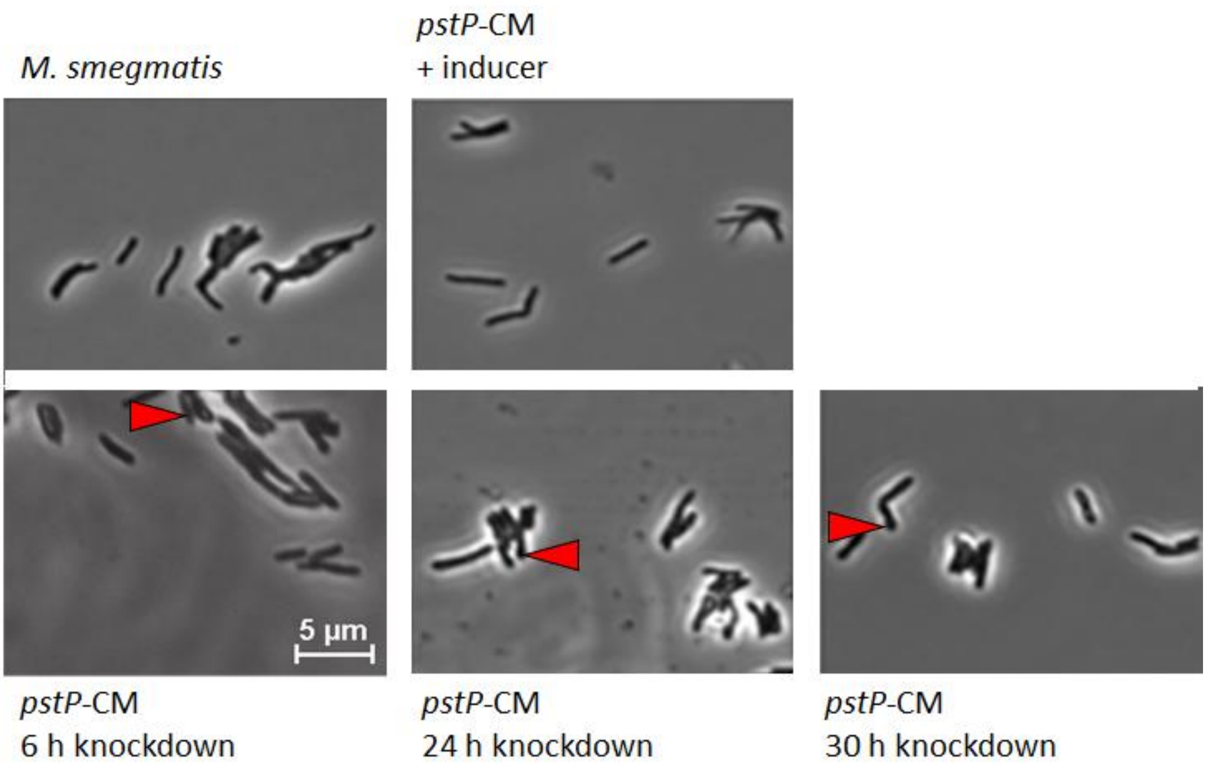

Figure S4. Knockdown of the *pstP* operon led to bulging cells (indicated by red arrowheads).

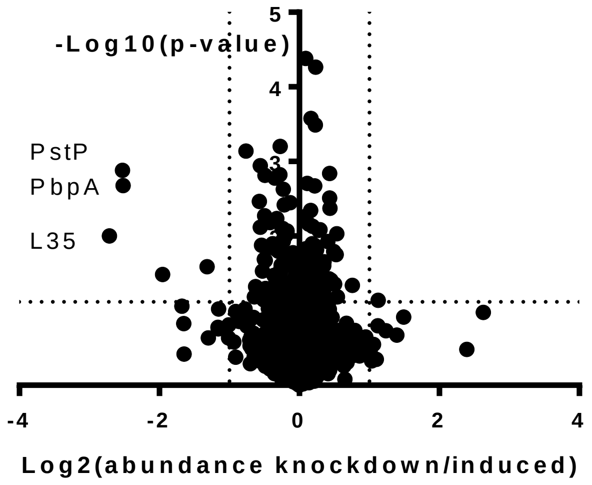

Figure S5. PstP and PbpA were depleted after six hours of knockdown of the *pstP* operon, but without global changes in protein concentration.

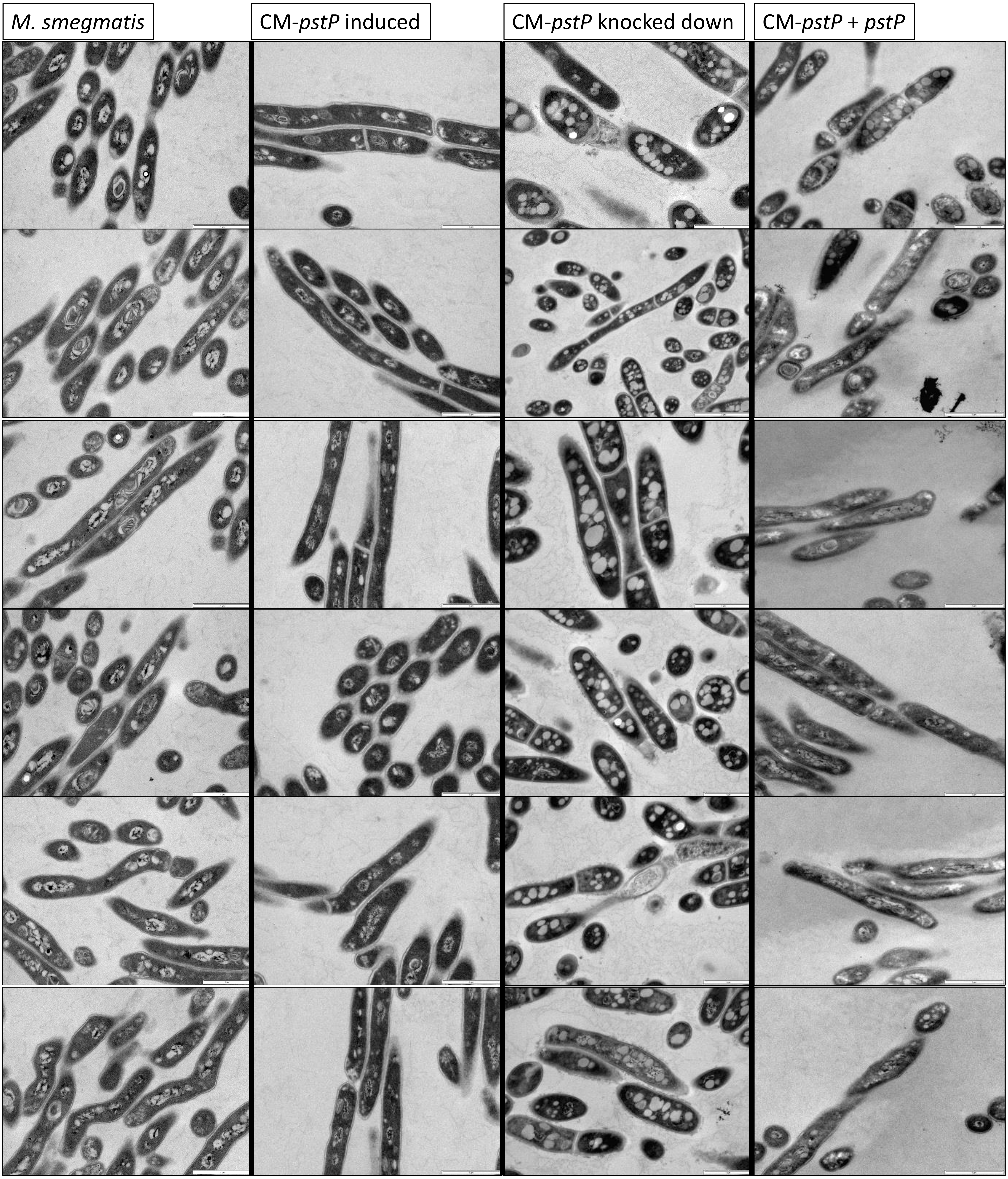

Figure S6. Additional transmission electron microscopy fields to accompany Figure 3. Panels show the presence/absence of lipid bodies, and smooth/rough surface of *M. smegmatis*, CM-*pstP* in induced conditions and after 30 hours knockdown, and CM-*pstP* with *pstP* reintroduced and inducer removed for 30 hours. These images are representative of >100 cells for each condition. Each scale bar indicates 1 μm.

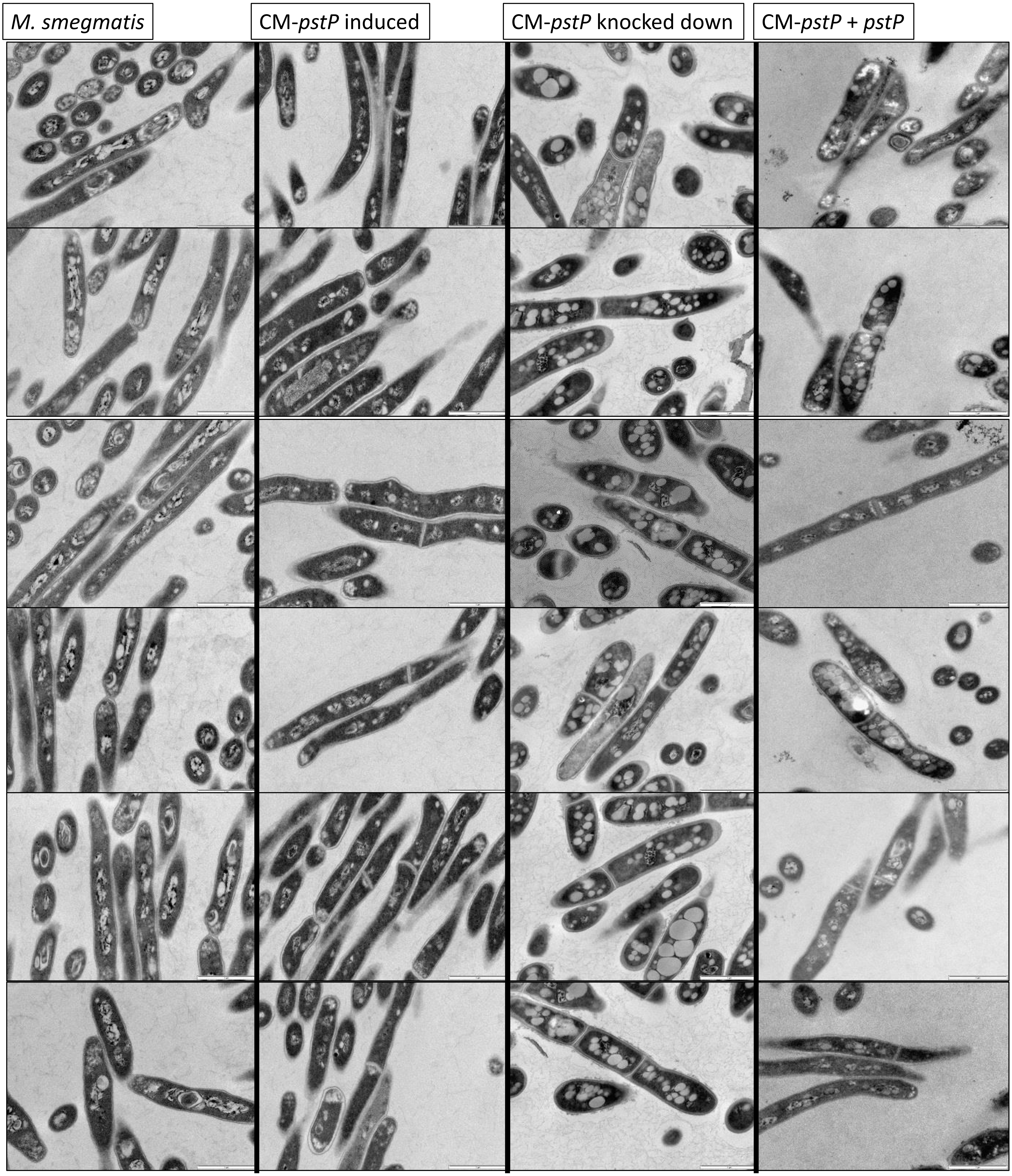

Figure S7. Additional transmission electron microscopy fields to accompany Figure 3. Panels show the thickness of the cell wall and septum of *M. smegmatis*, CM-*pstP* in induced conditions and after 30 hours knockdown, and CM-*pstP* with *pstP* reintroduced and inducer removed for 30 hours. These images are representative of >100 cells for each condition. Each scale bar indicates 1 μm.

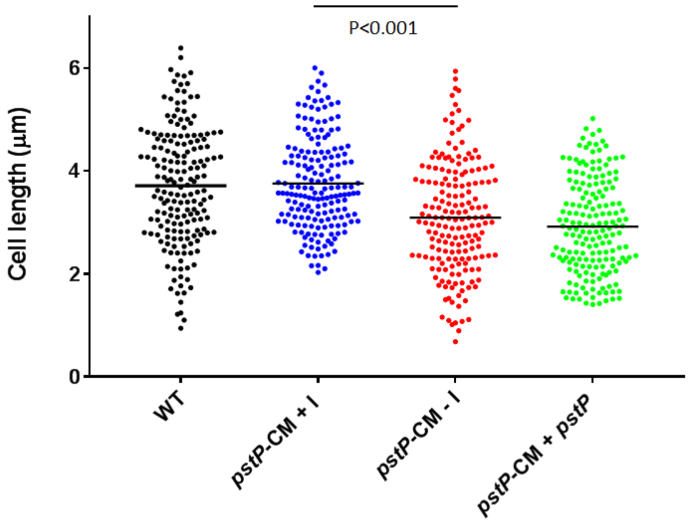

Figure S8. Cell length was reduced during knockdown of the *pstP* operon, and the length was not restored by reintroduction of *pstP*. Cell length was measured using SEM images of 100 cells. Abbreviation: I means inducer.

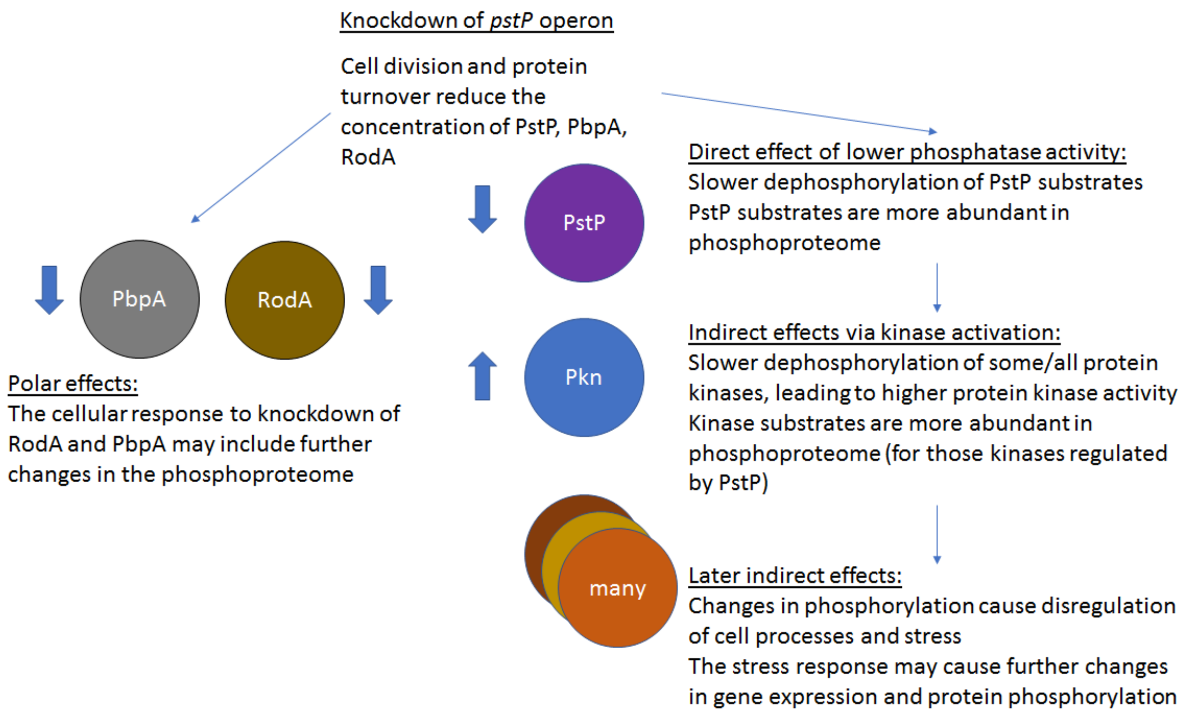

Figure S9. Direct and indirect mechanisms by which knockdown of the *pstP* operon may influence protein phosphorylation and cell physiology.
